# Saddlepoint p-values for a class of location-scale tests under randomized block design

**DOI:** 10.1038/s41598-024-53451-z

**Published:** 2024-02-07

**Authors:** Haidy N. Mohamed, Ehab F. Abd-Elfattah, Amel Abd-El-Monem, Abd El-Raheem M. Abd El-Raheem

**Affiliations:** Department of Mathematics, Faculty of Education, Ain Shams University, Cairo, Egypt

**Keywords:** Statistics, Computational biology and bioinformatics

## Abstract

This paper deals with a class of nonparametric two-sample location-scale tests. The purpose of this paper is to approximate the exact p-value of the considered class under a randomized block design. The exact p-value of the considered class is approximated by the saddlepoint approximation method, also by the traditional method which is the normal approximation method. The saddlepoint approximation method is more accurate than the normal approximation method in approximating the exact p-value, and does not take a lot of time like the simulation method. This accuracy is proved by applying the mentioned methods to two real data sets and a simulation study.

## Introduction

The tests for location-scale problem have many uses in many fields, especially when studying spatial and variations in streamflow, Zhang et al.^1^ and Yang et al.^2^, also in monsoon circulation, see Kwoon et al.^3^. In addition, these tests arise very often in bioinformatics in the case of comparing two groups such as control and treatment in order to detect differentially expressed genes, see Neuhäuser and Senske^4^. There is also an importance for location-scale tests in the field of biomedical and clinical trials, when the drug or the treatment is given for the treatment group causes changes in location and scale parameters between the two groups, see Mulccioli et al.^5^, Rice et al.^6^, Lunde and Timmermann^7^ and Marozzi^8^. This paper presents some location-scale linear rank tests that can form the proposed class. Lepage^9^ test “LP1”, was the first test that combines both, the location test which is the Wilcoxon test and the scale test which is the Ansari-Bradley test. In addition, Büning and Thadewald^10^ presented LP2, LP3 and LP4 tests which are known as modified tests for LP1. Furthermore, Rublik^11^ proposed a test statistic consisting of a linear combination of the location test which is the Wilcoxon test and the scale test which is the Mood test. Rousson^12^ discussed the location-scale test in case of a multivariate two-sample problem. All the aforementioned tests can be used when all observations in the population or selected samples are determined. Therefore, these tests belong to classical statistics. While if the data to be analyzed or a part of it is indeterminate, we can resort to neutrosophic statistics. For more information on this context, a number of references can be referred to, namely Smarandache^13^, Aslam^14–16^, Afzal et al.^17^, Albassam et al.^18^ and Sherwani et al.^19^. If you interested in location shift only, you could see Hollander et al.^20^. They suggested many location problems and tests, with one sample and two samples.

In clinical trials, consider we have $N$ patients will be distributed into two groups, control group and treatment group. To guarantee no selection bias in the randomization assignment for the subjects, and to achieve a certain degree of balance between the control and treatment groups, randomization designs must be used. Complete randomization design is one of the easiest ways to assign the subject into two groups using a fair coin, if the head, it goes to the control but if the tail, it goes to the treatment group, and so on until we finish all subjects. There is also a random allocation design, which depends on one of the $$\left(\begin{array}{c}N\\ \frac{N}{2}\end{array}\right)$$ permutations to assign the $N$ patients to the groups. The randomized block design is an important design that reduces selection bias and control the imbalance of group sizes. In this paper, we tend to use randomized block design, which contains $k$ blocks of even sizes ${n}_{i}$, such that $\frac{{n}_{i}}{2}$ patients are for control group and the same number for treatment group, and within each block the random allocation design is applied to assign the patients to the two groups. For more randomization designs, see Rosenberger and Lachin^21^.

To calculate the exact p-value of the proposed class, an accurate method is used, which is saddlepoint approximation method “SPA” that depends on the permutation distribution of the considered class. The SPA method discovered by Daniels^22^ who investigated an approximate formula for any probability mass or density function based on its moment generating function. The theory of SPA expansions and multidimensional generalizations were presented in Good^23,24^ and Barndorff-Nielsen and Cox^25^. Further developed by Lugannani and Rice^26^ who proposed an approximate formula for the cumulative distribution function, CDF. Saddelpoint approximations to randomization distributions and permutation distributions were treated by Robinson^27^ and Davison and Hinkley^28^. Skovgaard^29^ introduced an approximate formula for the conditional distribution function which is a generalization of the Lugannani and Rice^26^ formula, for more details, see Booth and Butler^30^ and Butler^31^. The SPA has many advantages, first, the SPA has a correction term equal to $O({n}^{-\frac{3}{2}} )$, in contrast the central limit theorem has $O({n}^{-\frac{1}{2}} )$, Daniels^32^. Second, the SPA often leads to a uniformly bounded error, in contrast, the errors of the normal approximation method generally increase in the tail of the distribution. Daniels^33^ was the first who suggested the major advantages of the SPA method and its general power and scope. Many papers were interested in approximating the exact p-value for various classes using SPA method with different randomization designs, such as, Abd-Elfattah and Butler^34,35^ Abd-Elfattah^36–39^, Abd EL-Raheem and Abd-Elfattah^40,41^, Kamal et al.^42,43^ and Abd El-Raheem et al.^44,45^.

The exact distribution of the considered class of tests is unknown. Then, we cannot obtain the exact p-values for such tests. Therefore, we resort to the saddlepoint approximation method to approximate the exact p-values for such tests. In all previous studies related to the considered class of tests, the normal approximation method was used to approximate the exact p-values of such tests. In the current study, we use the saddlepoint approximation method to approximate the exact p-value for the considered tests. From the results of simulation study and real data analysis, it will be seen that saddlepoint approximation p-values are almost always closer to the simulated (permutation) p-values than the normal approximation. The degree of greater accuracy is readily apparent in small and intermediate size samples for which the asymptotic normality has not been attained. Thus, the saddlepoint method is a more accurate approximation method than the normal approximation method and computationally less demanding than the simulation method (permutation based, so time consuming).

This paper is partitioned as follows: “Class of location-scale tests” presents the class of non-parametric location-scale tests. The saddlepoint approximation is presented in “Saddlepoint approximation”. Section “Simulation study and real data examples” proves the accuracy of SPA in approximating the exact p-value by performing a simulation study and analyzing three real data sets. Moreover, the time consumed for calculating the SPA p-values, normal approximation p-values and simulated mid-p-values is calculated in minutes and presented in “Simulation study and real data examples”. Furthermore, the 95% and 99% confidence intervals for location and scale parameters are constructed in “Confidence intervals for location and scale parameters”. Finally, the conclusion and discussion are presented in “Conclusion”.

## Class of location-scale tests

Consider two independent samples $X$ which is the control group and $Y$ is the treatment group are drawn from populations with CDF $F$ and $G$, with means ${\mu }_{1}$ and ${\mu }_{2}$ and standard deviations ${\sigma }_{1}$ and ${\sigma }_{2}$, respectively. Under the randomized block design, the location-scale class is given by

1 $${H}_{B}=\sum_{i=1}^{k}{b}_{i}\sum_{j=1}^{{n}_{i}}{(a}_{{L}_{ij}}+{a}_{{S}_{ij}}){Z}_{ij}, i=1,\dots ,k, j=1,\dots ,{n}_{i},$$

where $k$ is the number of blocks with even sizes ${n}_{i}$, $N=\sum_{i=1}^{k}{n}_{i}$, and ${b}_{i}=\frac{1}{{n}_{i}+1}$ is the optimum weight of block $i$, Elteren^46^. The location and scale scores of each observation $j$ in each block $i$ are donated by the linear combination ${(a}_{{L}_{ij}}+{a}_{{S}_{ij}} )$, where ${a}_{{L}_{ij}}$ is for location score and ${a}_{{S}_{ij}}$ is for scale score, and ${Z}_{ij}$ is the group indicator takes the value 1 if the observation $j$ in the block $i$ is from the treatment group $Y$ and takes the value 0 otherwise. The permutation distribution of the observations assignments within the blocks, is done under random allocation rule with $$\left(\begin{array}{c}{n}_{i}\\ {m}_{i}\end{array}\right)$$ possible permutations, where ${m}_{i}$ is the number of the treatment observations inside the block $i$. The asymptotic distribution of ${H}_{B}$ is $N\left( {\mu }_{L}+{\mu }_{S},{\sigma }_{L}+{\sigma }_{S}\right)$, where ${\mu }_{L}$ and ${\mu }_{S}$ are the means of the location and scale tests, respectively. Also, ${\sigma }_{L}\mathrm{ and }{\sigma }_{S}$ are the standard deviations of the location and scale tests, respectively.

The considered class includes many of location-scale tests, such as, Lepage’s statistic which can be written according to the Eq. (1) as

2 $$LP1=\sum_{i=1}^{k}{b}_{i}\sum_{j=1}^{{n}_{i}}{(W}_{{L}_{ij}}+{R}_{{S}_{ij}}){Z}_{ij},$$

where ${W}_{{L}_{ij}}$ is the score of the Wilcoxon location test with mean ${\mu }_{L}=\frac{1}{2}{\sum_{i=1}^{k}{b}_{i}m}_{i}\left({n}_{i}+1\right)$ and variance ${\sigma }_{L}^{2}=\frac{1}{12}{\sum_{i=1}^{k}{b}_{i}}^{2}{m}_{i}^{2}\left({n}_{i}+1\right).$ Also, ${R}_{{S}_{ij}}$ is the score of Ansari-Bradley scale test that takes the form

$${R}_{{S}_{ij}}=\mathrm{1,2},\mathrm{3,4}, \dots ,\frac{{n}_{i}}{2},\frac{{n}_{i}}{2},\dots ,\mathrm{4,3},\mathrm{2,1},$$

with mean

$${\mu }_{S}=\frac{1}{4}{\sum_{i=1}^{k}{b}_{i}{m}_{i}\left({n}_{i}+2\right)},$$

and variance

$${\sigma }_{S}^{2}=\sum_{i=1}^{k}\frac{{ {b}_{i}^{2}m}_{i}^{2}\left({n}_{i}^{2}-4\right)}{48\left({n}_{i}-1\right)},$$

with asymptotic distribution $N\left( {\mu }_{L}+{\mu }_{S},{\sigma }_{L}+{\sigma }_{S}\right)$. In addition, $LP2$, also called Gastwirth^47^ test, can be written in the form of Eq. (1), as follows:

3 $$LP2=\sum_{i=1}^{k}{b}_{i}\sum_{j=1}^{{n}_{i}}{(a}_{{L}_{ij}}+{a}_{{S}_{ij}}){U}_{ij},$$

where the location score is

$$a_{{L_{{ij}} }} = \left\{ {\begin{array}{*{20}l} {j - \frac{{n_{i} + 1}}{4}} \hfill & {if\;j \le \frac{{n_{i} + 1}}{4}} \hfill \\ 0 \hfill & {if\frac{{n_{i} + 1}}{4} < j < \frac{{3\left( {n_{i} + 1} \right)}}{4}} \hfill \\ {j - \frac{{3\left( {n_{i} + 1} \right)}}{4}} \hfill & {if\;j \ge \frac{{3\left( {n_{i} + 1} \right)}}{4},} \hfill \\ \end{array} } \right.$$

and the scale score

$$a_{{S_{{ij}} }} = \left\{ {\begin{array}{*{20}l} {\frac{{n_{i} + 1}}{4} - j} \hfill & {if\;j \le \frac{{n_{i} + 1}}{4}} \hfill \\ 0 \hfill & {if\frac{{n_{i} + 1}}{4} < j < \frac{{3\left( {n_{i} + 1} \right)}}{4}} \hfill \\ {j - \frac{{3\left( {n_{i} + 1} \right)}}{4}} \hfill & {if\;j \ge \frac{{3\left( {n_{i} + 1} \right)}}{4},} \hfill \\ \end{array} } \right.$$

where

$$U_{{ij}} = \left\{ {\begin{array}{*{20}l} 1 \hfill & {Z_{{i(j)}} \in Y} \hfill \\ 0 \hfill & {otherwise,} \hfill \\ \end{array} } \right.$$

and $Z_{{i(j)}}$ is the *j*-th order statistic of the combined two samples $X$ and $Y$ in the block $i$*.*

$LP3$ test takes the form of $LP2$ in Eq. (3) and follows the form of location-scale class in Eq. (1) with location score of Van der Waerden test

$${a}_{{L}_{ij}}={\varphi }^{-1}\left(\frac{j}{{n}_{i}+1}\right),$$

and Klotz scale score

$${a}_{{S}_{ij}}={\left({\varphi }^{-1}\left(\frac{j}{{n}_{i}+1}\right)\right)}^{2},$$

where ${\varphi }^{-1}$ is the inverse cumulative distribution function of standard normal distribution.

$LP4$ test also takes the same form of $LP2$ and $LP3$ but with location score

$${a}_{{L}_{ij}}=\left\{\begin{array}{c}-\left(\left[\frac{{n}_{i}}{4}\right]+1\right) if j<\left(\left[\frac{{n}_{i}}{4}\right]+1\right) \\ i-\frac{{n}_{i}+1}{2} if \left(\left[\frac{{n}_{i}}{4}\right]+1\right)\le j\le \left[\frac{3\left({n}_{i}+1\right)}{4}\right]\\ \left[\frac{{n}_{i}}{4}\right]+1 if j>\left[\frac{3\left({n}_{i}+1\right)}{4}\right],\end{array}\right.$$

and with Mood scale score

$${{a}_{S}}_{ij}={\left(j-\frac{{n}_{i}+1}{2}\right)}^{2},$$

where [*a*] denotes the greatest integer less than or equal to *a*.

All Lepage’s types, $LP2, LP3,{\text{and}} LP4$ are asymptotically distributed $N\left( \mu ,\sigma \right),$ where

$$\mu =\sum_{i=1}^{k}\frac{{{b}_{i}m}_{i}}{{n}_{i}}\sum_{j=1}^{{n}_{i}}{(a}_{{L}_{ij}}+{a}_{{S}_{ij}}),$$

and

$${\sigma }^{2}=\sum_{i=1}^{k}\frac{{{b}_{i}^{2}m}_{i}^{2} }{\left({n}_{i}-1\right)}\sum_{j=1}^{{n}_{i}}{\left({(a}_{{L}_{ij}}+{a}_{{S}_{ij}})-\frac{\sum_{j=1}^{{n}_{i}}{(a}_{{L}_{ij}}+{a}_{{S}_{ij}})}{{n}_{i}}\right)}^{2}.$$

In addition, Rublik^11^ investigated the location-scale problem with test statistic contains a combination between the Wilcoxon location score and the Mood scale score, that can take the same form of (1) as follows:

$$T=\sum_{i=1}^{k}{b}_{i}\sum_{j=1}^{{n}_{i}}\left(j+{\left(j-\frac{{n}_{i}+1}{2}\right)}^{2}\right){Z}_{ij},$$

with location mean ${\mu }_{L}=\frac{1}{2}\sum_{i=1}^{k}{b}_{i}{m}_{i} \left({n}_{i}+1\right),$ and variance ${\sigma }_{L}^{2}=\frac{1}{12}{{\sum_{i=1}^{k}{b}_{i}}^{2}m}_{i}^{2}\left({n}_{i}+1\right)$, and scale mean ${\mu }_{S}=\frac{1}{12}\sum_{i=1}^{k}{b}_{i}{m}_{i}\left({n}_{i}^{2}-1\right)$ and variance ${\sigma }_{S}^{2}=\frac{1}{180 }{{\sum_{i=1}^{k}{b}_{i}}^{2}m}_{i}^{2}\left({n}_{i}+1\right)\left({n}_{i}^{2}-4\right).$ The asymptotic distribution of $T$ is $N\left( {\mu }_{L}+{\mu }_{S},{\sigma }_{L}+{\sigma }_{S}\right)$.

In this paper, we are interested in working with three test statistics only, which are $LP1$ test, $LP3$ test and Rublik test. For more location-scale tests, see Duran et al.^48^ who investigated a class of location-scale nonparametric tests. Also see, Fueda and $\widehat{{\text{O}}}$hori^49^ who designed a two-sample rank test based on the Wilcoxon test.

## Saddlepoint approximation

For simplicity, let the class (Eq. 1) be in the form

4 $${H}_{B}=\sum_{i=1}^{k}\sum_{j=1}^{{n}_{i}}{A}_{ij}{Z}_{ij}, i=1,\dots ,k, j=1,\dots ,{n}_{i},$$

where ${{{A}_{ij}=b}_{i}(a}_{{L}_{ij}}+{a}_{{S}_{ij}})$, as we noted before, that the patients within the blocks distributed under random allocation design, this means that the random variables ${Z}_{ij}$ and ${Z}_{ib}$ for all $i=1,\dots ,k$ and $j\ne b$ are dependent but independent with ${Z}_{aj}$ where $a\ne i$.

To avoid the problem of the dependence, we constructed a conditional distribution as follow:

$${Z}_{11,}\dots , {Z}_{k{n}_{k}} \sim {V}_{11},\dots , {V}_{k{n}_{k}} | \sum_{j=1}^{{n}_{1}}{V}_{1j}={m}_{1},\dots ,\sum_{j=1}^{{n}_{k}}{V}_{kj}={m}_{k},$$

where ${V}_{i1}, \dots , {V}_{i{n}_{i}}$ are independent and identically Bernoulli (${\theta }_{i}$) random variables for each $i=1,\dots ,k$.

This transfers the distribution of the statistic (Eq. 4) to equivalent conditional distribution as follows:

$$\sum_{i=1}^{k}\sum_{j=1}^{{n}_{i}}{A}_{ij}{Z}_{ij}\sim \sum_{i=1}^{k}\sum_{j=1}^{{n}_{i}}{A}_{ij}{V}_{ij}| \sum_{j=1}^{{n}_{1}}{V}_{1j}={m}_{1},\dots ,\sum_{j=1}^{{n}_{k}}{V}_{kj}={m}_{k}.$$

Now, to approximate exact p-value of ${H}_{B}$ in (Eq. 4), we need to approximate the following conditional probability

5 $$P\left(\sum_{i=1}^{k}\sum_{j=1}^{{n}_{i}}{A}_{ij}{V}_{ij}\ge {h}_{o}| \sum_{j=1}^{{n}_{1}}{V}_{1j}={m}_{1},\dots ,\sum_{j=1}^{{n}_{k}}{V}_{kj}={m}_{k}\right),$$

where ${h}_{o}$ is the observed value of ${H}_{B}$, using the double saddlepoint approximation of Skovgaard^29^, the conditional probability in (Eq. 5) can be approximated as follows:

$${\text{midp}}\left({h}_{o}\right)\simeq 1-\Phi \left(\widehat{\omega }\right)-\phi \left(\widehat{\omega }\right)\left(\frac{1}{\widehat{\omega }}-\frac{1}{\widehat{u}}\right),$$

where

$$\widehat{\omega }=sgn\left(\widehat{t}\right)\sqrt{2\left[\left\{Q\left(0,{\widehat{S}}_{0}\right)-{{M}^{T}\widehat{S}}_{0}\right\}-\left\{Q\left(\widehat{t},\widehat{S}\right)-{M}^{T}\widehat{S}-{h}_{o}\widehat{t}\right\}\right]},$$

$$\widehat{u}=\widehat{t}\sqrt{\left|{Q}^{{\prime}{\prime}}\left(\widehat{t},\widehat{S}\right)\right|/\left|{Q}_{ss}^{{\prime}{\prime}}\left(0,{\widehat{S}}_{0}\right)\right|},$$

where $M=({m}_{1},\dots , {m}_{k})$, the two saddlepoints are $\left(\widehat{t},\widehat{S}\right)=\left(\widehat{t},{\widehat{s}}_{1},\dots , {\widehat{s}}_{k}\right)$ and ${\widehat{S}}_{0}=({\widehat{s}}_{10},\dots ,{\widehat{s}}_{k0})$.

The joint cumulant generating function of $\left\{\sum_{i=1}^{k}\sum_{j=1}^{{n}_{i}}{A}_{ij}{V}_{ij}, \sum_{j=1}^{{n}_{1}}{V}_{1j}={m}_{1}, \dots ,\sum_{j=1}^{{n}_{k}}{V}_{kj}={m}_{k}\right\}$ is

$$Q\left(t,S\right)=\sum_{i=1}^{k}\sum_{j=1}^{{n}_{i}}\mathit{ln}\left\{(1-{\theta }_{i})+{\theta }_{i}\mathit{exp}\left({s}_{i}+t{(a}_{{L}_{ij}}+{a}_{{S}_{ij}})\right)\right\},$$

where $Q\left(\widehat{t},\widehat{S}\right)$ is $(k+1)\times (k+1)$ Hessian matrix and ${Q}_{ss}^{{\prime}{\prime}}$ is the second derivative of $Q\left(0,{\widehat{S}}_{0}\right)$ with respect to $S$. To calculate $\widehat{\omega }$ and $\widehat{u}$, we first calculate the numerator saddlepoints $\left(\widehat{t},\widehat{S}\right)=\left(\widehat{t},{\widehat{s}}_{1},\dots , {\widehat{s}}_{k}\right)$, by solving the following equations

$${Q}_{{S}_{i}}{\prime}\left(\widehat{t},\widehat{S}\right)=\sum_{i=1}^{N}\frac{{\theta }_{i}\mathit{exp}\left({s}_{i}+t{(a}_{{L}_{ij}}+{a}_{{S}_{ij}})\right)}{(1-{\theta }_{i})+{\theta }_{i}\mathit{exp}\left({s}_{i}+t{(a}_{{L}_{ij}}+{a}_{{S}_{ij}})\right)}={m}_{i}, i=1,\dots ,k,$$

$${Q}_{t}{\prime}\left(\widehat{t},\widehat{S}\right)=\sum_{i=1}^{k}\sum_{j=1}^{{n}_{i}}\frac{{\theta }_{i}\mathit{exp}\left({s}_{i}+t{(a}_{{L}_{ij}}+{a}_{{S}_{ij}})\right){(a}_{{L}_{ij}}+{a}_{{S}_{ij}})}{(1-{\theta }_{i})+{\theta }_{i}\mathit{exp}\left({s}_{i}+t{(a}_{{L}_{ij}}+{a}_{{S}_{ij}})\right)}={h}_{o},$$

and to find the value of ${\widehat{S}}_{0}=\left({\widehat{s}}_{10},\dots ,{\widehat{s}}_{k0}\right),$ we solve the following equation

$$Q_{{s_{i} }} \prime \left( {0,\hat{S}_{0} } \right) = \sum\limits_{{j = 1}}^{{n_{i} }} {\frac{{\theta _{i} exp\left( {\hat{s}_{{i0}} } \right)}}{{(1 - \theta _{i} ) + \theta _{i} exp\left( {\hat{s}_{{i0}} } \right)}}} = m_{i} ,i = 1, \ldots ,k.$$

Each of $\widehat{\omega }$ and $\widehat{u}$ dose not depend on ${\theta }_{i}$, so for explicitly in solving the SPA equations, we can choose ${\theta }_{i}=\frac{{m}_{i}}{{n}_{i}}$, then ${\widehat{S}}_{0}=(0,\dots ,0)$ for all $i=1,\dots ,k$.

## Simulation study and real data examples

### Simulation study

The main aim of using a simulation study is to prove that the saddlepoint approximation method is closer to the simulated mid-p-value than the normal approximation method. The exact p-values of Lepage test, LP3 test and Rublik test are approximated using the two methods, saddlepoint approximation and normal approximation methods. To illustrate the accuracy of SPA p-value, we compare the p-values for the two previously methods with the simulated mid-p-value, which can be calculated from simulation of one million random permutations of the treatment and control indicator ${Z}_{ij}$. The simulated mid-p-value is obtained as $[\sum I(H>{h}_{0})+0.5\sum I(H={h}_{0})]/{10}^{6}$, and its donated here as “exact” p-value. The benefit of using mid-p-value instead of the p-value is that the mid-p-value is convenient in case of discrete test statistics and not conservative compared to the ordinary p-value, for more details, see Agresti^50^, Butler^31^ and Delanchy et al.^51^. The simulated data in this section is generated from extreme value and logistic distributions with six cases which are (1): $N=24,k=4,m=3$, case (2): $N=30,k=3,m=5$, case (3): $N=40, k=4, m=5$, case (4): $N=60, k=6, m=5$, case (5): $N=80, k=5, m=8$ and case (6): $N=90, k=5, m=5,$ each with ${n}_{i}=\frac{N}{k}$, and ${m}_{i}=m.$ Different location $({\mu }_{1},{\mu }_{2}$) and scale (${\sigma }_{1}, {\sigma }_{2}$) parameters are used to generate the data according to the previous scenarios. To prove our aim this process is repeated 1000 times based on 1000 generated samples. Tables 1, 2 and 3 present the mean of the SPA p-values, normal approximation p-values, simulated mid-p-values and the percentage of approaching the SPA method to the simulated method “P.SPA”. Also, the average relative absolute error for SPA “R.E.SPA” and the average relative absolute error for normal approximation method “R.E.NA” are calculated and presented in Tables 1, 2 and 3.

**Table 1:** Outcomes of the simulation study for the Lepage test.

Lepage test						
Cases	$N$ = *24,* $k$ = *4,* $m$ = *3*	$N$ = *30,* $k$ = *3,* $m$ = *5*	$N$ = *40,* $k$ = *4,* $m$ = *5*	$N$ = *60,* $k$ = *6,* $m$ = *5*	$N$ = *80,* $k$ = *5,* $m$ = *8*	$N$ = *90,* $k$ = *9,* $m$ = *5*
Extreme value distribution						
${\mu }_{1},{\mu }_{2}, {\sigma }_{1},{\sigma }_{2}$	0, 1, 3, 2	3, 1, 1, 2	0, 3, 3, 3.5	0, 1, 3, 2	0, 1, 0.1, 1.5	0, 1, 3, 2
Exact p-value	0.2442	0.0202	0.0454	0.1382	0.0221	0.1085
SPA p-value	0.2426	0.0202	0.0452	0.1380	0.0221	0.1084
Normal p-value	0.2416	0.0207	0.0453	0.1378	0.0222	0.1084
P.SPA	80.1	95.6	84.1	76.0	86.1	73.4
R.E.SPA	0.0298	0.0004	0.0476	0.0216	0.1497	0.0253
R.E.NA	0.0601	0.0010	0.4318	0.0616	0.5431	0.0764

Logistic distribution						
${\mu }_{1},{\mu }_{2}, {\sigma }_{1},{\sigma }_{2}$	3, 1, 5, 10	0, 3, 1, 1.5	0, 3, 1, 2	3, 1, 5, 10	0, 3, 3, 2	3, 1, 5, 10
Exact p-value	0.2661	0.0306	0.0640	0.1586	0.0094	0.1090
SPA p-value	0.2649	0.0301	0.0635	0.1586	0.0094	0.1089
Normal p-value	0.2642	0.0304	0.0635	0.1585	0.0095	0.1089
P.SPA	81	85.3	82.0	77.5	89.6	74.6
R.E.SPA	0.0061	0.0917	0.0482	0.0019	0.1196	0.0007
R.E.NA	0.0110	1.7408	0.4445	0.0025	0.4601	0.0010

**Table 2:** Outcomes of the simulation study for the $LP3$ test.

LP3 test						
Cases	$N$ = *24,* $k$ = *4,* $m$ = *3*	${\text{N}}$ = 30, ${\text{k}}$ = 3, ${\text{m}}$ = 5	${\text{N}}$ = 40, ${\text{k}}$ = 4, ${\text{m}}$ = 5	${\text{N}}$ = 60, ${\text{k}}$ = 6, ${\text{m}}$ = 5	${\text{N}}$ = 80, ${\text{k}}$ = 5, ${\text{m}}$ = 8	$N$ = *90,* $k$ = *9,* $m$ = *5*
Extreme value distribution						
${\upmu }_{1},{\upmu }_{2}, {\upsigma }_{1},{\upsigma }_{2}$	1, 0, 0.1, 1	3, 0, 5, 1	3, 0, 1, 2	1, 0, 0.1, 1	3, 0, 50, 10	1, 0, 0.1, 1
Exact p-value	0.5370	0.0025	0.1277	0.3897	0.0003	0.3677
SPA p-value	0.5366	0.0024	0.1273	0.3895	0.0003	0.3676
Normal p-value	0.5367	0.0035	0.1270	0.3893	0.0004	0.3675
P.SPA	81.7	99.4	93.8	91.6	99.3	85.1
R.E.SPA	0.0086	6.2 $\times {10}^{-5}$	0.0008	0.0055	7.6 $\times {10}^{-6}$	0.0091
R.E.NA	0.0205	0.0011	0.0034	0.0423	6.6 $\times {10}^{-5}$	0.0390

Logistic distribution						
${\upmu }_{1},{\upmu }_{2}, {\upsigma }_{1},{\upsigma }_{2}$	0, 2, 5, 15	3, 0, 50, 10	3, 0, 50, 10	0, 2, 5, 15	3, 0, 5, 7	0, 2, 5, 15
Exact p-value	0.2043	0.0940	0.0725	0.0539	0.4974	0.0241
SPA p-value	0.2033	0.0934	0.0721	0.0537	0.4974	0.0240
Normal p-value	0.2026	0.0934	0.0723	0.0539	0.4974	0.0243
P.SPA	83.5	89.2	94.2	92.5	89.3	91.9
R.E.SPA	0.0202	0.0009	0.0005	0.0387	0.0024	0.0765
R.E.NA	0.2401	0.0037	0.0023	0.5868	0.0097	0.5964

**Table 3:** Outcomes of the simulation study for the Rublik test.

Rublik test						
Cases	$N$ = *24,* $k$ = *4,* $m$ = *3*	$N$ = *30,* $k$ = *3,* $m$ = *5*	$N$ = *40,* $k$ = *4,* $m$ = *5*	$N$ = *60,* $k$ = *6,* $m$ = *5*	$N$ = *80,* $k$ = *5,* $m$ = *8*	$N$ = *90,* $k$ = *9,* $m$ = *5*
Extreme value distribution						
${\mu }_{1},{\mu }_{2}, {\sigma }_{1},{\sigma }_{2}$	1, 3, 1, 2	1, 3, 0.1, 0.01	1, 3, 0.1, 0.5	1, 3, 1, 2	0, 3, 1, 2	1, 3, 1, 2
Exact p-value	0.0737	0.0478	0.0268	0.0488	0.0853	0.0210
SPA p-value	0.0733	0.0481	0.0266	0.0487	0.0853	0.0210
Normal p-value	0.0735	0.0481	0.0274	0.0488	0.0850	0.0212
P.SPA	65.7	84.6	81.9	82.7	71.7	83.8
R.E.SPA	0.0465	0.0067	0.0086	0.0155	0.0052	0.0307
R.E.NA	0.1524	0.0080	0.0220	0.0807	0.0091	0.1262

Logistic distribution						
${\mu }_{1},{\mu }_{2}, {\sigma }_{1},{\sigma }_{2}$	0, 0.01, 1, 0.5	0, − 0.1, 1, 1.5	0, 0.01, 1, 0.5	0, 0.1, 1, 1.5	0, 0.001, 1.5, 1	0, 0.01, 1, 0.5
Exact p-value	0.2644	0.2600	0.1138	0.0906	0.1147	0.0486
SPA p-value	0.2627	0.2597	0.1135	0.0904	0.1146	0.0485
Normal p-value	0.2619	0.2588	0.1133	0.0904	0.1144	0.0486
P.SPA	76	81.9	83.1	82.6	81.2	81.1
R.E.SPA	0.0064	0.0094	0.0009	0.0005	0.0004	0.0003
R.E.NA	0.0098	0.0436	0.0019	0.0011	0.0008	0.0005

In Table 1, 2 and 3, the SPA approximation is more accurate than the normal approximation method. This can be seen through the R.E.SPA, which is much smaller than R.E.NA, for all considered cases.

### Real data examples

To support the aim of this paper, three real data sets are analyzed. The first data set is from Rosenberger and Lachin^21^. They analyzed cholesterol rate for 50 patients. The results of the cholesterol rate for 50 patients can be found in Table 7.4 in the reference Rosenberger and Lachin^21^. The 50 patients were assigned randomly to control and treatment group by generating the vector of the group indicator $${Z}_{ij}$$, such that 25 assign to control and 25 assign to treatment, where each block contains 5 from each group, i.e. ($$N=50,{n}_{i}=10 ,k=5, m=5$$). The second data set is from a survey of household expenditure for 20 single men” treatment group” and 20 single women ”control group”. For this data set, ($$N=40,{n}_{i}=8, k=5, m=4)$$. The second data is presented in Büning and Thadewald^10^. The third data set was presented on page 39 of Hand et al.^52^. This data set consists of 40 measurements of cholesterol levels for 40 men were divided into two groups A and B according to two types of behaviors. The type A behavior “treatment group” is characterized by urgency and aggression. While type B behavior “control group” is relaxed. For this data set, ($$N=40,{n}_{i}=8, k=5, m=4)$$. Table 4 presents the p-values of $$LP1$$ test, $$LP3$$ test and Rublik test using simulated, SPA and normal approximation methods.

**Table 4:** P-values for simulated, SPA and normal approximations for the three data sets.

Data sets	LP1			LP3			Rublik		
	SPA	Exact	Normal	SPA	Exact	Normal	SPA	Exact	Normal
1st	0.2397	0.2403	0.2381	0.2767	0.2773	0.2733	0.2705	0.2705	0.2710
2nd	0.3655	0.3681	0.3638	0.0135	0.0138	0.0152	0.0093	0.0094	0.0104
3rd	0.0128	0.0130	0.0137	0.1216	0.1219	0.1192	0.3934	0.3911	0.3975

From Table 4, we can see that SPA p-value is closer to the exact p-value than the normal p-value, and this result gives more evidence that SPA method is more accurate than the normal method in approximating the p-value. It remains for us to explain the reason for considering the saddlepoint approximation method as an alternative to the simulation method. The reason is that the saddlepoint approximation method requires much less computing time compared to the simulation method. To clarify this, the computing time for the different methods is calculated and this is summarized in Tables 5, 6 and 7.

**Table 5:** The time consumed for calculating the SPA p-values, normal approximation p-values and simulated mid-p-values for Lepage test.

Lepage test						
Cases	$N$ = *24,* $k$ = *4,* $m$ = *3*	$N$ = *30,* $k$ = *3,* $m$ = *5*	$N$ = *40,* $k$ = *4,* $m$ = *5*	$N$ = *60,* $k$ = *6,* $m$ = *5*	$N$ = *80,* $k$ = *5,* $m$ = *8*	$N$ = *90,* $k$ = *9,* $m$ = *5*
Extreme value distribution						
${\mu }_{1},{\mu }_{2}, {\sigma }_{1},{\sigma }_{2}$	0, 1, 3, 2	3, 1, 1, 2	0, 3, 3, 3.5	0, 1, 3, 2	0, 1, 0.1, 1.5	0, 1, 3, 2
Exact p-value	1504 min	1615 min	1613 min	1701 min	1617 min	1713 min
SPA p-value	5.13 min	5.14 min	6.12 min	5.62 min	5.23 min	5.33 min
Normal p-value	3.41 min	3.52 min	3.44 min	4.11 min	3.21 min	3.21 min

Logistic distribution						
${\mu }_{1},{\mu }_{2}, {\sigma }_{1},{\sigma }_{2}$	3, 1, 5, 10	0, 3, 1, 1.5	0, 3, 1, 2	3, 1, 5, 10	0, 3, 3, 2	3, 1, 5, 10
Exact p-value	1515 min	1512 min	1527 min	1621 min	1629 min	1723 min
SPA p-value	5.71 min	5.21 min	5.32 min	6.22 min	6.10 min	6.30 min
Normal p-value	3.21 min	3.01 min	3.22 min	3.41 min	3.41 min	3.22 min

**Table 6:** The time consumed for calculating the SPA p-values, normal approximation p-values and simulated mid-p-values Rublik test.

Rublik test						
Cases	$N$ = *24,* $k$ = *4,* $m$ = *3*	$N$ = *30,* $k$ = *3,* $m$ = *5*	$N$ = *40,* $k$ = *4,* $m$ = *5*	$N$ = *60,* $k$ = *6,* $m$ = *5*	$N$ = *80,* $k$ = *5,* $m$ = *8*	$N$ = *90,* $k$ = *9,* $m$ = *5*
Extreme value distribution						
${\mu }_{1},{\mu }_{2}, {\sigma }_{1},{\sigma }_{2}$	1, 3, 1, 2	1, 3, 0.1, 0.01	1, 3, 0.1, 0.5	1, 3, 1, 2	0, 3, 1, 2	1, 3, 1, 2
Exact p-value	1603 min	1613 min	1502 min	1528 min	1619 min	1624 min
SPA p-value	5.21 min	5.66 min	5.24 min	5.44 min	5.55 min	5.36 min
Normal p-value	2.99 min	3.12 min	2.97 min	3.25 min	3.75 min	3.19 min

Logistic distribution						
${\mu }_{1},{\mu }_{2}, {\sigma }_{1},{\sigma }_{2}$	0, 0.01, 1, 0.5	0, − 0.1, 1, 1.5	0, 0.01, 1, 0.5	0, 0.1, 1, 1.5	0, 0.001, 1.5, 1	0, 0.01, 1, 0.5
Exact p-value	1533 min	1630 min	1623 min	1525 min	1522 min	1623 min
SPA p-value	6.00 min	6.25 min	6.61 min	5.43 min	5.31 min	5.29 min
Normal p-value	3.24 min	3.55 min	3.76 min	3.42 min	3.46 min	3.12 min

**Table 7:** The time consumed for calculating the SPA p-values, normal approximation p-values and simulated mid-p-values for LP3 test.

LP3 test						
Cases	$N$ = *24,* $k$ = *4,* $m$ = *3*	${\text{N}}$ = 30, ${\text{k}}$ = 3, ${\text{m}}$ = 5	${\text{N}}$ = 40, ${\text{k}}$ = 4, ${\text{m}}$ = 5	${\text{N}}$ = 60, ${\text{k}}$ = 6, ${\text{m}}$ = 5	${\text{N}}$ = 80, ${\text{k}}$ = 5, ${\text{m}}$ = 8	$N$ = *90,* $k$ = *9,* $m$ = *5*
Extreme value distribution						
${\upmu }_{1},{\upmu }_{2}, {\upsigma }_{1},{\upsigma }_{2}$	1, 0, 0.1, 1	3, 0, 5, 1	3, 0, 1, 2	1, 0, 0.1, 1	3, 0, 50, 10	1, 0, 0.1, 1
Exact p-value	1051 min	1060 min	1072 min	1075 min	1067 min	1173 min
SPA p-value	6.20 min	6.54 min	5.61 min	6.43 min	6.21 min	6.49 min
Normal p-value	3.34 min	3.35 min	3.16 min	3.22 min	3.16 min	3.92 min

Logistic distribution						
${\upmu }_{1},{\upmu }_{2}, {\upsigma }_{1},{\upsigma }_{2}$	0, 2, 5, 15	3, 0, 50, 10	3, 0, 50, 10	0, 2, 5, 15	3, 0, 5, 7	0, 2, 5, 15
Exact p-value	1054 min	1057 min	1162 min	1167 min	1267 min	1263 min
SPA p-value	5.00 min	5.45 min	5.21 min	5.42 min	6.31 min	6.31 min
Normal p-value	3.23 min	3.16 min	3.77 min	3.52 min	3.86 min	3.97 min

From the result of the simulation study, we can see that the SPA method is more accurate than the normal approximation methods compared to the simulated exact p-value. Moreover, from Tables 5, 6 and 7 it is clear that the SPA method is faster than simulated method which needs a lot of time to approximate the exact p-value.

## Confidence intervals for location and scale parameters

The estimated confidence intervals for location parameter ${\mu }_{2}$ and scale parameter ${\upsigma }_{2}$, are the set of all values ${\mu }_{{2}_{o}}$ and ${\upsigma }_{{2}_{{\text{o}}}}$ of the parameters ${\mu }_{2}$ and ${\upsigma }_{2}$, respectively, which if formulated in the claim ${H}_{o}: {\mu }_{2}={\mu }_{{2}_{o}}$ and ${\upsigma }_{2 }={\upsigma }_{{2}_{{\text{o}}}}$, would not be rejected at α significant level. Accordingly, if $p\left({\mu }_{{2}_{o}}, {\upsigma }_{{2}_{{\text{o}}}}\right)$ is the one-sided p-value for the location-scale test, then a $\left(1-\alpha \right)100\%$ confidence intervals of ${\mu }_{2}$ and ${\upsigma }_{2}$ can be constructed as $\left\{{\mu }_{{2}_{o}}:\frac{\alpha }{2}\le p\left({\mu }_{{2}_{o}}, {\upsigma }_{{2}_{{\text{o}}}}\right)\le 1-\frac{\alpha }{2}\right\}$ and $\left\{{\upsigma }_{{2}_{{\text{o}}}}:\frac{\alpha }{2}\le p\left({\mu }_{{2}_{o}}, {\upsigma }_{{2}_{{\text{o}}}}\right)\le 1-\frac{\alpha }{2}\right\}$, respectively, see^34^. Assume ${D}_{o }\left({\mu }_{{2}_{o}}, {\upsigma }_{{2}_{{\text{o}}}}\right)$ be the observed test statistic with location parameter ${\mu }_{{2}_{o}}$ and scale parameter ${\upsigma }_{{2}_{{\text{o}}}}$, using a satisfactory grid of ${\mu }_{{2}_{o}}$ and ${\upsigma }_{{2}_{{\text{o}}}}$ values with suitable increasing, the cutoff ${D}_{o }\left( ., .\right)$ is a step function in ${\mu }_{{2}_{o}}$ and ${\upsigma }_{{2}_{{\text{o}}}}$ that leads to incremental increases with increasing ${\mu }_{{2}_{o}}$ and ${\upsigma }_{{2}_{{\text{o}}}}$. Here, the 3^rd^ real data set is used to illustrate the procedure for creating confidence intervals for the location and scale parameters. We use the “gofTest” R package to estimate the location ${\mu }_{2}$ and scale ${\upsigma }_{2}$ parameters for the 3^rd^ real data set and to test the suitability of the extreme value distribution for the considered real data set. The maximum likelihood estimations for the location and scale parameters are 227.9 and 31.17, respectively. Furthermore, the p-value of the goodness of fit test, is *p*-value = 0.887. We evaluate the values of ${D}_{o }\left({\mu }_{{2}_{o}}, {\upsigma }_{{2}_{{\text{o}}}}\right)$ using a large range of the possible values of ${\mu }_{{2}_{o}}{\text{and}} {\upsigma }_{{2}_{{\text{o}}}}$, then for each value of ${D}_{o }\left({\mu }_{{2}_{o}}, {\upsigma }_{{2}_{{\text{o}}}}\right)$ the corresponding exact, saddlepoint and normal p-values are calculated. Table 8 includes the exact, saddlepoint and normal confidence intervals for LP1 test.

**Table 8:** 99% and 95% confidence intervals for the location and scale parameters of the 3rd data set for LP1 test.

	Location parameter	Scale parameter
99% Confidence interval		
Exact	(151.4, 308.4)	(29.34, 111.6)
SPA	(151.4, 308.4)	(29.34, 111.6)
Normal	(146.9, 311.9)	(29.23, 115.1)
95% Confidence interval		
Exact	(158.4, 319.4)	(29.51, 122.6)
SPA	(158.4, 319.4)	(29.51, 122.6)
Normal	(158.4, 319.4)	(29.51, 122.6)

From Table 8, we can see that the estimated 99% confidence interval using SPA method is more accurate than the corresponding estimated confidence interval using the normal approximation method as compared to the simulated (Exact) confidence interval. For the 95% confidence interval, both methods have the same accuracy as the simulated method.

## Conclusion

In this article, various nonparametric tests for location and scale problem have been discussed and rewritten as a common linear rank class. The exact p-value of the considered class is approximated by SPA method and the normal approximation method. According to our results in the simulation study and the two real data sets, SPA performs well and achieves high accuracy in approximating the exact p-value instead of the normal method. This article can be applied in different designs, such as random allocation design, Wei’s urn design, complete design and truncated binomial design. Also, the proposed study can be extended to neutrosophic statistics, see Afzal et al.^17^, Albassam et al.^18^ and Sherwani et al.^19^.

## Data Availability

The datasets used and/or analyzed during the current study available from the corresponding author on reasonable request.
